# The antioxidant and anti-cadmium toxicity properties of garlic extracts

**DOI:** 10.1002/fsn3.164

**Published:** 2014-10-06

**Authors:** Suwannaporn Boonpeng, Sunisa Siripongvutikorn, Chutha Sae-wong, Pornpong Sutthirak

**Affiliations:** 1 Department of Functional Food and Nutrition, Faculty of Agro-Industry, Prince of Songkla UniversityHat Yai, Songkhla, 90112, Thailand; 2 Department of Food Technology, Faculty of Agro-Industry, Prince of Songkla UniversityHat Yai, Songkhla, 90112, Thailand; 3 Aquatic Animal Biotechnology Research Center, Faculty of Science and Industrial Technology, Prince of Songkla UniversityMueang, Suratthani, 84000, Thailand

**Keywords:** Anti-Cd, antioxidant, garlic, pickled garlic

## Abstract

Cadmium (Cd) contamination is a highly dangerous international problem because it can transfer into the food chain and become bioaccumulated, endangering human health. Cd detoxication is very interesting particularly the method providing no undesirable side effects. Cd also causes lipid oxidation that leads to undesired food quality. Garlic (*Allium sativum* L.) has been used as conventional food and in herbal therapy and folklore medicine as an antibacterial, antitumorogenic, and antioxidant agent for over 5000 years. In the present work, fresh garlic and pickled garlic extracted with distilled water was brought to determine antioxidant activities in terms of 1,1-diphenyl-2-picrylhydrazyl (DPPH) radical scavenging assay, 2,2′-azino-*bis*-3-ethylbenzthiazoline-6-sulphonic acid (ABTS) radical scavenging assay, ferric reducing ability power (FRAP) assay, chelating activities, superoxide, and hydroxyl scavenging assay. The data showed that pickled garlic extracts significantly possessed more DPPH, ABTS, FRAP, superoxide, and hydroxyl scavenging assays as 11.86, 13.74, 4.9, 46.67, and 15.33 g trolox equivalent/g sample, respectively, compared with fresh one as 7.44, 7.62, 0.01, 4.07, and 8.09 g trolox equivalent/g sample, respectively. However, iron chelating activity of fresh garlic extract was higher than that of pickled garlic while there was no significant difference in the copper chelating activity of both extracts. For anti-Cd properties, pickled garlic was more effective than fresh garlic and contained less toxicity than standard diallyl disulfide (DADS). Therefore, therapeutic properties of pickled garlic favored its consumption compared with fresh and standard DADS for its antioxidant and anti-Cd properties.

## Introduction

Cadmium (Cd) contamination in the environment is a subject of serious international recognition as metals could enter the food chain and bioaccumulate, therefore endangering human health (Morkmek et al. 2010). Industries that manufacture products such as electroplating, plastic production, pigments, battery manufactures, and pesticides are the main source of Cd contamination. Additionally, vegetable origin products are the major carriers of Cd compounds in food (Pari et al. 2007). Food is the main source of Cd intake for nonoccupationally exposed people. According to the World Health Organization (WHO), the limit of tolerable intake for Cd is 7 *μ*g/kg bw/week (Agency for Toxic Substances and Disease Registry 2008).

The main pathway of Cd exposure to humans is by ingestion of Cd in water or food and by inhalation of fumes or particles during industrial operations (Morkmek et al. 2010). Cd generally accumulates in the liver and the kidney. Cd-induced cytotoxicity is closely related to the persuasion of oxidative stress. These evidences imply that apoptosis possibly plays an important role in acute and chronic intoxication with Cd (Pari et al. 2007). Cd toxicity is related to its interaction with carboxyl and thiol groups of proteins and is able to generate free radicals during induced oxidative stress. However, living cells possess diverse mechanisms to maintain free metal concentrations at levels that do not exceed cellular requirements. One of the best described mechanisms against heavy metal toxicity is found in some yeasts such as algae, photosynthetic protists, and plants that involves their intracellular chelation by either glutathione (GSH) or phytochelatins (PCs), low-molecular-weight sulfur-containing peptides derived from GSH, or both.

GSH is found in all organisms participating in multiple metabolic processes, for example, intracellular redox state regulation, inactivation of reactive oxygen species (ROS), transport of GSH-conjugated amino acids and other molecules, and storage of sulfur and cysteine affording up to 90% of the nonprotein sulfur in the cell. GSH synthesis, starting from inorganic sulfate, requires the sulfur assimilation pathway (SAP) and the cysteine biosynthetic (Cys) pathway (Mendoza-Cόzatl et al. 2005). Thus, sulfur is an important factor in GSH synthesis for reducing toxicity of Cd. Although many chelating agents and antagonists have been found to reduce Cd toxicity, part of them reveal undesirable side effects. Because of the intrinsic limitations and variability in the efficacy of heavy metal chelating agents, Cd detoxication is eagerly awaiting development of novel generation therapeutic agents with various modes of actions, especially from medicinal plants throughout the garlic clove generally used in food and medicine. The unique organosulfur compounds (OSCs) in garlic are believed to play key biological roles. Many studies showed that garlic's rich OSCs reveal diverse biological activity, including antitumorogenic, antimutagenic, antioxidant detoxification, and other activities (Murugavel et al. 2007).

Suru (2008) and Obioha et al. (2009) reported that Cd-induced nephrotoxicity and hepatoprotection in rats significantly reduced and increased, respectively, when using aqueous garlic extract. As a result, the levels of renal and liver lipid peroxidation (LPO) and glutathione-*S*-transferase (GST) significantly increased (*P *<* *0.001) and the level of GSH, superoxide dismutase (SOD), catalase (CAT), and Na^+^/K^+^-ATPase significantly decreased (*P *<* *0.001) compared with the control which received Cd alone. However, the result also showed that the rats treated with a high dose of garlic elicited a pro-oxidant effect, relative to their respective low dose. While Pari et al. (2007) investigated the cytoprotective and antioxidant role of diallyl tetrasulfide (DTS) on Cd-induced renal injury in the kidney of rats and also in kidney cell line (vero cell). The result showed that the kidney function of rats that received 40 mg/kg bw/day of DTS and 3 mg/kg bw/day of Cd showed a significant decrease in LPO and an increase in the antioxidant defense system. The vero cell treated with DTS at 40 *μ*g/mL could block the LPO and cell death induced by 10 *μ*mol/L Cd, indicating its cytoprotective property. Moreover, Murugavel et al. (2007) determined the protective effect on Cd of garlic DTS that induced apoptosis and oxidative stress in vero cells. Vero cells treated with Cd (10 *μ*mol/L) and DTS (5–50 *μ*g/mL) indicated that DTS decreased the Cd-induced suppression of cell viability in a dose-dependent manner and the effect was highly significant at 40 *μ*g/mL. DTS at a concentration of 40 *μ*g/mL extremely decreased the Cd-induced accumulation of hydrogen peroxide and superoxide radical within cells. Moreover, DTS significantly prevented the Cd-induced decrease in mitochondrial membrane potential, an indicator of mitochondrial function. Massadeh et al. (2007) stated that reduction of Cd accumulation in the liver, kidney, heart, spleen, and blood were 72.5%, 87.7%, 92.6%, 95.6%, and 71.7%, respectively, when 12.5–100 mg/L of garlic extract was applied to the rats.

Many OSCs in garlic are produced by readjustment of allicin resulting in the production of diallyl sulfide (DAS), diallyl disulfide (DADS), diallyl trisulfide (DTS), dithiins, and ajoene depending on condition. One gram of fresh garlic consists of 2.5 mg of allicin and about 500 *μ*g of DADS or DTS. In fact, allicin and other thiosulfinates have a short shelf-life in aqueous solution, while DADS, ajoene, and dithiines are lipid-soluble compounds and are more stable (Kay et al. 2010). Moreover, it has been proved that allicin, an unstable compound, will be decomposed to DADS (66%), DAS (14%), DTS (9%), and sulfur dioxide (Amagase 2006). DADS was reported to increase the level of the phase II detoxification enzyme GSH transferase in various parts of the gastrointestinal tract (Robert et al. 2001). José et al. (2003) reported that DADS had a protective effect on oxidative stress and antioxidant activity of enzymes in rat's kidney treated with an overdose of gentamicin (GM) (70 mg/kg/12 h/4 days). Moreover, DADS increased the GSH level in Cd-treated cell line (Lawal and Elizabeth 2011).

Garlic is consumed in several forms such as bulbs, crushed or chopped, and pickled. The pickling process of garlic generally aims to extend shelf life and reduce the strong odor of garlic as well as generate a new product. Pickled garlic, Gratiem Dong (Thai), is frequently used in many Thai dishes. It is used to provide a salty, sour, and rich flavor to dishes. Moreover, pickled garlic or gratiem dong have been added in many Thai salads and soups. For making pickled garlic, root and stem of the fresh garlic are cut, the bulbs are then washed and drained before they are soaked in a pickled solution which consists of vinegar, sugar, and salt. However, the recipe for pickled garlic may differ from producer to producer in terms of the ingredients used in the pickled solution or the steps involved in the manufacturing of pickled garlic.

Thai garlic is smaller and has a stronger smell compared with Chinese garlic (Chinawong 2000). It is believed that pickled garlic processing in Thailand is also different than the methods employed in other countries. So, utilizing the research information obtained from other regions might not be very reliable. Therefore, fresh and pickled Thai garlic were monitored for their antioxidant and anti-Cd activities.

## Material and Methods

Thai garlic grown in Lumphoon province, the northern part of Thailand, from the same field and lot was used as raw material for fresh and commercial pickled garlic. After harvesting time, the fresh garlic bulb was pickled according to procedures adopted by small and medium enterprises (SMEs). Both fresh and pickled garlic were analyzed for moisture content (AOAC 1995), pH and *a*_w_ following the method of AOAC (1990).

### Chemical

Human embryonic kidney cells (HEK 293) were purchased from American Type Culture Collection. Chemicals were used for determination of anti-Cd toxicity (Gibco® and Invitrogen, Carlsbad, CA). Most of the chemicals used for determination of antioxidant activity were purchased from Sigma-Aldrich, Seelze, Germany (otherwise from Merck, Darmstadt, Germany; Ajax Finechem, Auckland, New Zealand; QRAC, Selangor, Malaysia; Fisher Scientific, Leicestershire, England; and LAB-SCAN, Dublin, Ireland.

### Preparation of garlic extracts

Fresh garlic bulbs were peeled, washed, chopped, and homogenized with distilled water at a ratio of 1:2 (garlic:distilled water) and then stirred for 12 h at room temperature before subjected to filter through cheesecloth and centrifuged at 6000*g* (Avanti® J-E; Beckman Coulter, TX). The clear supernatant was collected and freeze-dried. The commercial pickled garlic was washed with tap water twice to remove the excess pickled solution and then drained for 2 min before brought to peel, wash, chop, homogenize and extract as mentioned in fresh garlic extract.

### Preparation of DADS solution

DADS was purchased from Sigma-Aldrich chemie GmbH, Steinheim, Germany and dissolved with 0.1% dimethyl sulfoxide (DMSO) to obtain concentrations of 50, 100, 200, and 400 *μ*mol/L or 7.31, 14.63, 29.26, and 58.51 *μ*g/mL, respectively, which were used as standard agent.

### Antioxidant activities

#### ABTS (Re et al. 1999)

3-Ethylbenzthiazoline-6-sulphonic acid (ABTS) was dissolved in water to obtain a 4 mmol/L concentration. ABTS radical cation (ABTS^**·**+^) was prepared by reacting ABTS stock solution with 2.5 mmol/L potassium persulfate (at a ratio 1:1) and kept in the dark at room temperature for 12–16 h before used. Thereafter, 20 *μ*L of the sample at various concentrations (1–50 mg/mL) was added to 280 *μ*L of the ABTS^**·**+^ solution. The reaction mixture was incubated in a dark room for 2 h prior to absorbance measurement at 734 nm. The antioxidant activity of the sample is shown in terms of gram equivalents of Trolox (TE) per gram of sample.

#### FRAP assay (Benzie and Strain 1996)

Reagents included 300 mmol/L acetate buffer, pH 3.6 (3.1 g C_2_H_3_NaO_2_·3H_2_O and 16 mL C_2_H_4_O_2_ per liter of buffer solutions 10 mmol/L TPTZ (2,4,6-tripyridyl-*S*-triazine) in 40 mmol/L HCl (BDH Laboratory Supplies, Poole, England); 20 mmol/L FeCl_3_·6H_2_O (BDH Laboratory Supplies). Working ferric reducing ability power (FRAP) reagent was prepared by mixing 25 mL acetate buffer, 2.5 mL TPTZ solution, and 2.5 mL FeCl_3_·6H_2_O solution. The solution was incubated in a water bath at 37°C for 30 min. Then, 30 *μ*L (1–50 mg/mL) of each sample was added to the FRAP reagent and incubated in the dark room for 30 min prior to absorbance measurement at 595 nm. The antioxidant activity of the sample is expressed in terms of gram equivalents of TE per gram of sample.

#### Copper-chelating activity

The Cu^2+^-chelating activity was determined using pyrocatechol violet (PV) according to Sánchez-Vioque et al. (2012). Two milliliters of 50 mmol/L sodium acetate buffer pH 6.0 and 50 *μ*L of 5 mmol/L CuSO_4_ were added to the samples at 1–50 mg/mL (0.5 mL). After incubation at room temperature for 30 min, 50 *μ*L of 4 mmol/L PV was added. After 30 min, the absorbance was measured at 632 nm. Distilled water and Ethylenediaminetetraacetic acid (EDTA) were used as control and as a standard metal chelator of the assay, respectively. The chelating activity of the sample was expressed in terms of gram equivalents of EDTA per gram of sample.

#### Iron chelating activity (Decker and Welch 1990)

Two hundred microliters of different concentrations of the sample (1–50 mg/mL) was mixed with 20 mL of FeCl_2_ solution (1.2 mmol/L in H_2_O). Ferrozine (2.4 mmol/L, 20 *μ*L) was incorporated and the mixtures were incubated for 10 min. The absorbance was measured at 562 nm. The chelating activity of a sample was expressed in terms of gram equivalents of EDTA per gram of sample.

#### DPPH radical scavenging activity assay (Brand-Williams et al. 1995)

1,1-Diphenyl-2-picrylhydrazyl (DPPH) was prepared at 0.2 mmol/L in absolute methanol. Exactly 100 *μ*L of the sample (1–50 mg/mL) was mixed with 100 *μ*L of DPPH in a 96-well microtiter plate. The reaction mixtures were incubated at room temperature for 30 min before absorbance measurement at 517 nm. The DPPH radical scavenging activity of sample was revealed in terms of gram equivalents of TE per gram of sample.

#### Superoxide scavenging assay (adapted from Su et al. 2009)

Briefly, 0.2 mL of different extract concentrations (1–50 mg/mL) was added to 5.7 mL of 50 mmol/L Tris–HCl buffer at pH 8.2. The mixture was incubated at 25°C for 10 min following by the addition of 0.2 mL of 6 mmol/L pyrogallol (25°C). The absorbance was measured every 30 sec at 320 nm until the reaction progressed to 5 min. The antioxidant activity of a sample was expressed in terms of gram equivalents of TE per grams of sample.

#### Hydroxyl radical scavenging activity (Zhang et al. 2009)

The reagents were made in a test tube in the following order: 0.4 mL KH_2_PO_4_–KOH buffer (pH 7.5), 0.1 mL sample solution with a concentration of 1–50 mg/mL and 0.1 mL of 1 mmol/L EDTA, 10 mmol/L H_2_O_2_, 60 mmol/L 2-deoxy-d-ribose, 2 mmol/L ascorbic acid, and 1 mmol/L FeCl_3_ (distilled water was used as a control). The whole solution was then incubated at 37°C for 1 h. Thereafter, the reaction was stopped by adding 1 mL of 20% TCA (Trichloroacetic acid). 1 mL of 1% thiobarbituric acid (TBA) was added in order to develop the color. The reaction tubes were then boiled in water for 15 min and then the reaction solution tubes were rapidly cooled to room temperature. The absorbance was measured at 532 nm. The activity of the sample was expressed as gram equivalents of TE per grams of sample.

### Cell culture

HEK293 cell lines (human embryonic kidney cells) were all grown in minimum essential medium (MEM), supplemented with 10% fetal bovine serum (FBS) and 1% penicillin–streptomycin. The cells were kept at 37°C in a humidified atmosphere of 5% CO_2_ and 95% air for analysis.

### Cell viability assay

Cell viability was determined by the MTT assay. The cells were treated with garlic extracts for 24 h and/or Cd for 24 h in a 96-well plate. One hundred microliters of MTT (0.5 mg/mL) was subjected to each well and then incubated at 37°C for 3 h. Thereafter, it was aspirated and DMSO (100 *μ*L) was added to each well to dissolve the formazan. The cell was incubated at 37°C for 10 min and the absorbance was read at 570 nm using a microplate spectrophotometer (Mosmann 1983).

Cell viability (%) was calculated using the following equation:

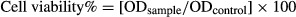


### Effect of cadmium chloride (CdCl_2_), fresh and pickled garlic extracts, and DADS on cell viability

Cell viability was measured after cells were grown following the cell culture method. Cells were plated in a 96-well plate at a density of 1 × 10^6^ cells/mL and allowed to attach for 24 h before treated with the samples as follows:

**Table:** 

Control	cells + 0, 50, 60, 70, 80, 90, 100, 110, 120, 130, 140, 150 *μ*mol/L CdCl_2_
Tested samples	cells + 0, 10, 25, 50, 100, 200, 400 *μ*g/mL fresh garlic extract cells + 0, 10, 25, 50, 100, 200, 400 *μ*g/mL pickled garlic extract
Standard	cells + 0, 10, 25, 50, 100, 200, 400 *μ*mol/L DADS

At least 80% of cell viability from three levels of the tested sample and standard agent was taken for the study. The Cd concentration gave 50% cell viability (IC_50_) of the control group that was selected.

### Anti-Cd toxicity properties on HEK293 cells

The protective effect of fresh and pickled garlic extracts and DADS on HEK293 cells induced by CdCl_2_ at 135.8 *μ*mol/L and % cell viability were investigated by the MTT assay. The cells were divided into three groups. After seeding in a 96-well plate at a density of 1 × 10^6^ cells/mL, the cells were allowed to attach for 24 h as follows:

Group 1: treated with the extracts and standard three levels for 24 h, then treated with 135.8 *μ*mol/L CdCl_2_ and incubated for 24 h to obtain 50% cell viability, IC_50_.
Group 2: treated with 135.8 *μ*mol/L CdCl_2_, the extracts and standard DADS together and incubated for 24 h.
Group 3: treated with 135.8 *μ*mol/L CdCl_2_, for 24 h, then treated with the extracts and standard and incubated for 24 h.

## Results

### Physical quality of fresh and pickled garlic extracts

The moisture content, water activity, and pH of fresh and pickled garlic are shown in Table1. The moisture content of fresh garlic was 64.37% lower than that of pickled garlic. This may due to the pickled process, but there was no significant difference in the water activity in both fresh and pickled garlic. The pH value of pickled garlic (3.89) was lower than that of fresh garlic (6.09).

**Table 1:** Moisture content, water activity, and pH value in fresh and pickled garlic

	Fresh garlic	Pickled garlic
Moisture content (%)	64.37 ± 0.15	84.57 ± 0.09
Water activity	0.99 ± 0.001	0.97 ± 0.001
pH value	6.09 ± 0.05	3.89 ± 0.27

Each value is expressed as mean ± SD (*n* = 3).

### Effect of fresh and pickled garlic extracts on antioxidant activities

Antioxidant activities of fresh and pickled garlic extracts were evaluated according to their proton donor, electron transfer, and metal chelating activity as determined by DPPH, ABTS, FRAP, iron, and copper chelating assays. The data showed that pickled garlic extracts significantly possessed more DPPH, ABTS, FRAP, superoxide, and hydroxyl (11.86, 13.74, 4.91, 46.67, and 15.33 × 10^−3^ g trolox equivalent/g sample, respectively) compared with fresh garlic (7.44, 7.62, 0.01, 4.07, and 8.09 × 10^−3^ g trolox equivalent/g sample, respectively). However, the iron chelating activity of the fresh extract was higher than that of the pickled one, while there was no significant difference in the copper chelating activity of both extracts (Table2).

**Table 2:** Antioxidant activities of fresh and pickled garlic extracts

Antioxidant activities	Gram trolox equivalent per gram sample	
	Fresh garlic	Pickled garlic
DPPH	7.44 ± 0.16^b^	11.86 ± 0.14^a^
ABTS	7.62 ± 0.32^b^	13.74 ± 0.14^a^
FRAP	0.01 ± 0.02^b^	4.91 ± 0.05^a^
Superoxide	4.07 ± 0.50^b^	46.67 ± 4.04^a^
Hydroxyl	8.09 ± 1.00 × 10^−3b^	15.33 ± 0.43 × 10^−3a^
	Gram EDTA equivalent per gram sample	
Iron chelating	0.61 ± 0.05^a^	ND^b^
Copper chelating	20.44 ± 0.17^a^	20.28 ± 0.28^a^

Each value is expressed as mean ± SD (*n* = 3). Means with different superscript letters within a row are significantly different (*P* < 0.05). ND, not detected.

### Effect of cadmium chloride (CdCl_2_), fresh and pickled garlic extracts, and DADS on cell viability

**Figure 1:**
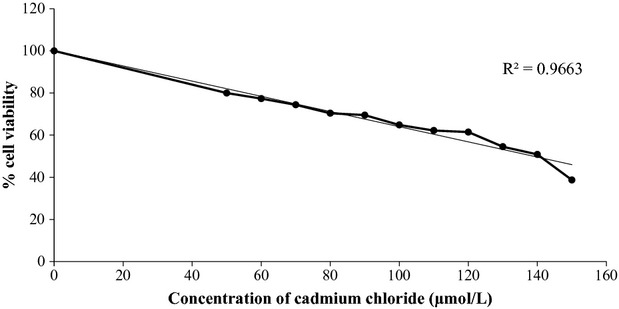
Effect of CdCl_2_ on cell viability determined using MTT assay.

**Figure 2:**
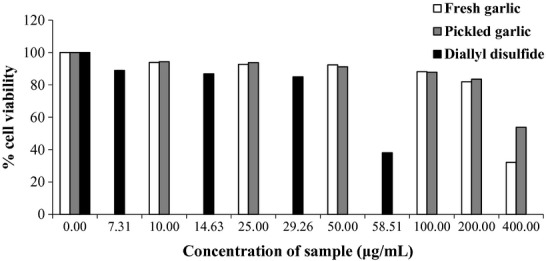
Effect of fresh and pickled garlic extracts and diallyl disulfide (μg/mL) on cell viability.

An increase in Cd concentration led to a decrease in cell viability with a strong correlation at *R*^2^ = 0.966 (Fig.1). It was found that the IC_50_ of CdCl_2_ was 135.8 *μ*mol/L (1.7 × 10^4^ *μ*g/mL). Both garlic extracts, fresh and pickled one, and DADS slightly exhibited cell toxicity (Fig.2). Although the extracts and DADS were applied to the cell at a concentration of 200 *μ*g/mL of garlic extracts and 29.26 *μ*g/mL (200 *μ*mol/L) of DADS, the cell viability still remained more than 80%. However, when 400 *μ*g/mL of the fresh garlic extract or 58.51 *μ*g/mL (400 *μ*mol/L) of DADS were applied, cell viability was significantly reduced and less than 50%, except the pickled garlic extract.

### Anti-Cd toxicity properties on HEK293 cells

**Figure 3:**
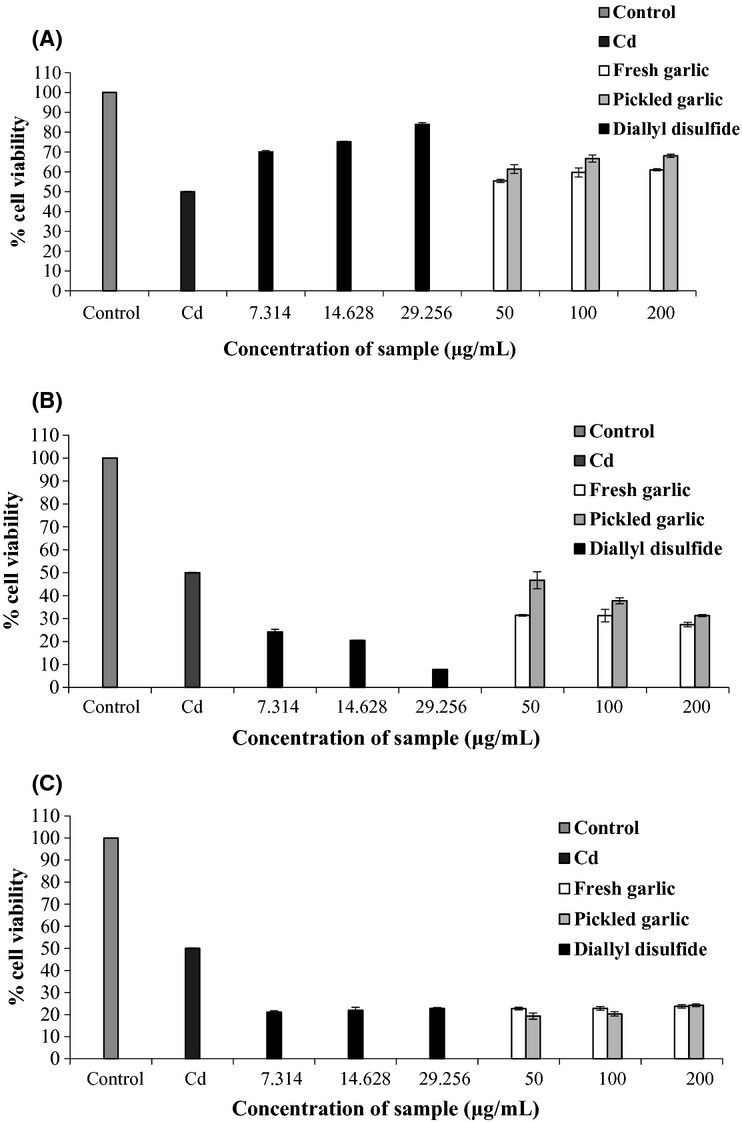
Anti-Cd toxicity properties on HEK293 cells determined using MTT assay. (A) The cells were treated with samples and standard before exposure Cd; (B) the cells were treated with samples, standard, and Cd together; and (C) the cells were exposed to Cd before treated samples and standard; each value is expressed as mean ± SD (*n* = 3).

Generally, cell viability was the highest (more than 50%) in the cell that was treated with the extracts and DADS before exposure to CdCl_2_ at a concentration 135.8 *μ*mol/L (Fig.3A) when compared with other treatments (Fig.3B,C). Moreover, cell viability significantly increased when the extracts and DADS increased (Fig.3A). Surprisingly, pickled garlic extract expressed high anti-Cd than fresh and DADS samples when treated CdCl_2_ in the sample in the same time of extracts or DADS (Fig.3B). In addition, higher DADS concentration caused more cell death compared with the extract samples (Fig.3B). It implied that pure DADS played a role in CdCl_2_ toxicity while at a lower concentration it turned out to be an anti-Cd agent and at a higher concentration it enhanced Cd toxicity. However, the injured cell when treated with CdCl_2_ did not recover by using the extracts or DADS (Fig.3C), suggesting that the extracts and DADS possessed a greater protective effect than curing effect.

## Discussion

### Physical quality of fresh and pickled garlic extracts

An increase in moisture content in pickled garlic may be caused by (1) different osmotic pressure between pickled solution and garlic cloves cell and/or (2) decrease in oil content and soluble solid due to leaching. The pH of pickled garlic was lower than that of fresh garlic due to the vinegar composition in the pickled solution (pH 3.71). Moreover, an increase in moisture content meant that there was a salted effect and/or some soluble solid was leached out.

### Effect of fresh and pickled garlic extracts on antioxidant activities

A comparison of electron transfer and/or hydrogen donor ability by using DPPH radical scavenging, ABTS, and FRAP activities showed that the activity of pickled garlic extract was the highest. Moreover, ABTS had higher value compared with DPPH radical scavenging and FRAP activities, implying the utility of ABTS antioxidant activity determination for hydrophilic and lipophilic compound measurement. The ABTS assay can be used to determine both hydrophilic and lipophilic compounds while the DPPH assay can be used to evaluate only less hydrophilic compounds. Moreover, the FRAP assay was used to analyze only the electron transfer property of the compound in acid condition (Wojdylo et al. 2007), suggesting that the active compound had donated electrons to reduce Fe^3+^ to Fe^2+^. Surprisingly, FRAP activity of the pickled extract was more than 400 times compared to that of the fresh extract. This may be due to different compounds occurring during pickling and/or acetic acid action as an antioxidant.

José et al. (2012) suggested that acetic acid was a weak acid and played a role as an antioxidant in black beans extraction by supporting the formation of hydrogen bonds during dissociation. The antioxidant capacity of acetic acid extract was higher than other organic solvent extracts determined by the DPPH assay. Kim et al. (2012a, 2012b) found that commercial vinegar drinks, particularly blackberry vinegar (BV) in Korea, were a potential source of antioxidants and the most generally occurring acid was acetic acid. The pH value of vinegar drinks ranged from 2.81 to 3.20. BV exhibited the highest antioxidant activity and red ginseng vinegar (HV) showed the lowest as determined by DPPH and ABTS activities.

Frankel et al. (1996) suggested that carnosol and carnosic acid derived from rosemary extract possessed higher antioxidant activity as determined by the inhibition of hydroperoxide formation at lower pH values (pH 4). Likewise, Juntachote and Berghofer (2005) reported that at neutral (pH 7) and acidic pH (pH 3 and 5), the ethanolic extracts of holy basil exhibited higher antioxidant activity as determined by superoxide anion scavenging activity, Fe^2+^ chelating activity, and reducing power. Thus, the higher antioxidative activity of pickled garlic extracts might be due to the formation of new compounds, acetic acid, and favorable conditions for antioxidant action when compared with fresh garlic extracts.

Additionally, superoxide scavenging and hydroxyl scavenging activities of the pickled extract were higher than the fresh one, around 10 and 2 times, respectively. After pickling, however, iron chelating activity could not be detected in the pickled extract, where as the copper chelating activity was similar when compared with the fresh extract. Results of this research indicate that pickled garlic seemed to contain higher antioxidant activities when compared with the fresh garlic. However, loss of iron chelating activity in pickled garlic may be due to the dissolving and/or leaching effect of the pickling solution. Generally, iron form in plants was chelated with ferritins, a class of multimeric proteins (Pich et al. 2001). Ferritins may be hydrolyzed, dissolved, or leached out when pickled, leading to lower iron chelating ability. The pickled extract seemed to be a pro-oxidant because upon mixing with ferrozine, the color changed to dark red and the OD was over 1 which was higher than that of the blank sample. However, this phenomenon should be investigated further.

Overall, antioxidant activities increased in pickled garlic. This result did not agree with the finding of Imai et al. (1994) who reported that allicin, a major active compound found in fresh garlic, played an important antioxidant role (DPPH) compared with *S*-allylcysteine (SAC), *S*-allylmercaptocysteine (SAMC) found in aged garlic, and alliin found in heat-treated garlic. However, Kim et al. (2012a, 2012b) stated that aged black garlic had higher antioxidant activity determined by DPPH, reducing power, and hydroxyl radical scavenging activities compared with fresh garlic, while Fe^2+^-chelating ability was considerably lower in aged black garlic than that of the fresh garlic extract. The controversial information mentioned above suggests that the nutritive value and antioxidant properties of fresh and pickled or aged garlic may differ. Moreover, garlic processed differently – heated, aged, or pickled – may give different results. This confirms that research information from different regions may differ due to raw material, process used, and method evaluated.

From the literature review, it was found that not only phenolic compounds and/or flavonoids can be natural antioxidants but also both vitamin E and C play a key role in antioxidant activity, particularly in the human body (Frankel 2007). Montaño et al. (2004) reported that the mean content of *α*-tocopherol (vitamin E) and ascorbic acid (vitamin C) of fresh garlic were 0.5 and 9.2 mg/100 g (wet basis, wt), respectively, while the content in pickled garlic were 1.3 and 14.7 mg/100 g (wt) (Casado et al. 2004). In this experiment both garlic forms (fresh and pickled) were good sources of antioxidant agents. Beato et al. (2012) reported that *S*-allyl-l-cysteine (SAC) increased by about 20% while *γ*-l-glutamyl-*S*-allyl-l-cysteine (GSAC), *γ*-l-glutamyl-*S*-(*trans*-1-propenyl)-l-cysteine (GSPC), and *γ*-l-glutamyl-*S*-methyl-l-cysteine (GSMC) decreased after pickling. Ana et al. (2012) reported that SAC had the ability to scavenge reactive oxygen (ROS) and nitrogen (RNS) species by increasing enzymatic and nonenzymatic antioxidant level, active Nrf2 factor, or inhibit some pro-oxidant enzyme (xanthine oxidase, cyclooxygenase, and NADPH oxidase). Maldonado et al. (2003) addressed that SAC protected the GM-induced oxidative stress and reduced manganese superoxide dismutase (Mn-SOD), glutathione peroxidase (GPx), and glutathione reductase (GR) activity in rats treated with 250 mg/kg SAC. Numagami and Ohnishi (2001) reported that SAC could decrease free radical and LPO as indicated by TBA-reactive substances (TBARS) in ischemic rat brain.

When superoxide and hydroxyl radical scavenging activities were determined through this experiment, it was found that consuming garlic as a natural antioxidant was highly beneficial to health. However, various previous studies have only dealt with antioxidant activities in terms of synthetic radicals. Therefore, using the results from in vitro antioxidant activity for health benefit prediction is still quite far from being realistic.

### Effect of cadmium chloride (CdCl_2_), fresh and pickled garlic extracts, and DADS on cell viability

During pickling, changes were observed for some active compounds and it is possible that DADS in pickled garlic might have reduced the content of toxicity compounds or changed them to nontoxic form.

### Anti-Cd toxicity properties on HEK293 cells

From the literature review, it was found that the kidney cell line (vero cell) treated with DTS at 40 *μ*g/mL effectively blocked the cell death and LPO induced by Cd (10 *μ*mol/L) indicating its cytoprotective property (Pari et al. 2007). And DTS (40 *μ*g/mL) remarkably decreased the Cd-induced accumulation of hydrogen peroxide and superoxide radical within cells. Furthermore, DTS significantly prevented the Cd-induced potential reduction in the mitochondrial membrane, a mitochondrial function indicator (Murugavel et al. 2007). Lawal and Elizabeth (2011) reported the efficacy of aged garlic extract (AGE). The AGE was extracted by soaking the crushed garlic into distilled water at room temperature for 1 year compared with DADS in preventing against toxicity induced by Cd in 1321N1 (human astrocytoma cells) and HEK293 (human embryonic kidney cells). The results showed that AGE (100 *μ*g/mL) could protect loss of cell viability in Cd-treated 1321N1 and HEK293 cells. On the contrary, DADS (100 *μ*mol/L) did not show any effect on the protection of HEK293 cells, but it did protect 1321N1 cells. AGE significantly decreased Cd-induced production of TBARS. Both AGE and DADS increased the levels of GSH in Cd-treated cell lines.

Figure3A to C shows that using pickled garlic extract could better protect injured cells when exposed to CdCl_2_ than fresh garlic and less toxicity compared with DADS. Therefore, it could be suggested that consuming pickled garlic provides greater health benefits than fresh garlic or pure DADS.

## Conclusion

Pickled garlic extract showed more potent scavenging radical activity than fresh garlic extract. However, fresh garlic seemed to have higher chelating activity than pickled garlic. As regards anti-Cd properties, pickled garlic was more effective than fresh garlic and less toxic than standard DADS. Therefore, due to its antioxidant and the anti-Cd properties, pickled garlic was a better alternative choice for consumption compared with fresh and standard DADS. However, the anti-Cd mechanism of pickled garlic, whether it could chelate or scavenge radical from Cd, has not been explained clearly, and it needs further study.
